# Cigarette Smoke Promotes Drug Resistance and Expansion of Cancer Stem Cell-Like Side Population

**DOI:** 10.1371/journal.pone.0047919

**Published:** 2012-11-05

**Authors:** Yi An, Alan Kiang, Jay Patrick Lopez, Selena Z. Kuo, Michael Andrew Yu, Eric L. Abhold, Jocelyn S. Chen, Jessica Wang-Rodriguez, Weg M. Ongkeko

**Affiliations:** 1 Division of Head and Neck Surgery, Department of Surgery, University of California San Diego, San Diego, California, United States of America; 2 Department of Pathology, School of Medicine, University of California San Diego, San Diego, California, United States of America; University of Barcelona, Spain

## Abstract

It is well known that many patients continue to smoke cigarettes after being diagnosed with cancer. Although smoking cessation has typically been presumed to possess little therapeutic value for cancer, a growing body of evidence suggests that continued smoking is associated with reduced efficacy of treatment and a higher incidence of recurrence. We therefore investigated the effect of cigarette smoke condensate (CSC) on drug resistance in the lung cancer and head and neck cancer cell lines A549 and UMSCC-10B, respectively. Our results showed that CSC significantly increased the cellular efflux of doxorubicin and mitoxantrone. This was accompanied by membrane localization and increased expression of the multi-drug transporter ABCG2. The induced efflux of doxorubicin was reversed upon addition of the specific ABCG2 inhibitor Fumitremorgin C, confirming the role of ABCG2. Treatment with CSC increased the concentration of phosphorylated Akt, while addition of the PI3K inhibitor LY294002 blocked doxorubicin extrusion, suggesting that Akt activation is required for CSC-induced drug efflux. In addition, CSC was found to promote resistance to doxorubicin as determined by MTS assays. This CSC-induced doxurbicin-resistance was mitigated by mecamylamine, a nicotinic acetylcholine receptor inhibitor, suggesting that nicotine is at least partially responsible for the effect of CSC. Lastly, CSC increased the size of the side population (SP), which has been linked to a cancer stem cell-like phenotype. In summary, CSC promotes chemoresistance via Akt-mediated regulation of ABCG2 activity, and may also increase the proportion of cancer stem-like cells, contributing to tumor resilience. These findings underscore the importance of smoking cessation following a diagnosis of cancer, and elucidate the mechanisms of continued smoking that may be detrimental to treatment.

## Introduction

Cigarette smoking is the largest risk factor for head and neck squamous cell carcinoma (HNSCC) and non-small cell lung carcinoma (NSCLC), common malignancies which together affect over 150,000 new patients in the US each year [1]. Chemical analysis shows that among the ∼4800 compounds found in cigarette smoke condensate (CSC), approximately 100 exhibit mutagenic activity [2]. Abundant within CSC are N-nitrosamines, such as 4-(methylnitrosamino)-1-(3-pyridyl)-1-butanone (NKK), and polyaromatic hydrocarbons, such as benzo[α]pyrene (B[α]P), which leads to formation of DNA adducts. All are believed to be major factors in smoking-induced carcinomas [3]. CSC-induced mutations in critical tumor-suppressor genes *p53 and p16* result in tumorigenesis [4]. Biochemical studies have also revealed that CSC can activate the pro-inflammatory protein NF-κB, along with proto-oncogenes such as ERK1/2, EGFR and Akt [5], [6], [7], [8].

Despite such effects of cigarette smoke, there is a lack of emphasis placed on smoking cessation during cancer treatment, due to the predominating view that treating tobacco dependence has little to no effect on treatment outcome. However, ongoing research suggests that continued smoking following a diagnosis may limit the effectiveness of treatment and increase the risk of mortality in some cancers. It was recently shown that smoking during radiation therapy was associated with unfavorable outcomes in head and neck cancer [9]. In addition, continued smoking during treatment of lung cancer leads to a higher incidence of recurrence, and is associated with a significantly greater risk of all-cause mortality [10], [11], [12]. Smoking cessation following a successful treatment of lung cancer was also associated with lower incidence of second primary cancers [12].

We speculated that cigarette smoke has a direct role in promoting chemoresistance, resulting in worsened treatment outcomes. In addition, the higher incidence of relapse associated with continued smoking may be an indication of cancer stem cell activity. We therefore turned our attention to the ATP-binding cassette transporter ABCG2/BCRP1, which plays an important role in drug resistance and has been used in some tissues as a stem cell marker. ABCG2 confers resistance towards multiple cytotoxic drugs, including topetcan, bisantrene, mitoxantrone, and doxorubicin, the last of which is commonly used to treat both HNSCC and NSCLC [13], [14]. ABCG2 expression is conserved among stem cells from a wide variety of origins and is also the molecular determinant of the side population, cells that show elevated efflux of Hoechst 33342 DNA-binding dye [15]. It has been shown that expression of ABCG2 in combination with CD133 predicts relapse in stage 1 NSCLC [16]. In esophageal squamous cell carcinoma, ABCG2 expression alone was associated with unfavorable prognoses [17].

The link between continued smoking and chemoresistance is unclear. Nicotine-induced upregulation of survivin and XIAP may protect cells against chemotherapy-induced death [18], but it is unknown whether cigarette smoke could promote chemoresistance by modulating drug transporter activity. Therefore, we sought to determine whether treatment with CSC enabled cells *in vitro* to increase ABCG2 expression and activity, and whether this change lead to enhanced cell viability in the presence of doxorubicin. We also examined whether CSC could expand the side population, which has been shown to possess cancer stem cell-like qualities [19]. The results of this study would help elucidate the role of tobacco use in cancer progression, leading to more effective treatment and management of smoking-related carcinomas.

## Materials and Methods

### Antibodies and Chemicals

Monoclonal mouse antibody against ABCG2 was purchased from Chemicon International (Temecula, CA). ß-Actin monoclonal mouse antibody was obtained from Sigma-Aldrich (St. Louis, MO). Rabbit p-Akt and mouse Akt antibodies were from Cell Signaling (Beverly, MA). PI3K inhibitor LY294002 was purchased from Calbiochem (San Diego, CA). Doxorubicin was obtained from Ortho Biotech (Bridgewater, NJ) and cigarette smoke condensate was obtained from Murty Pharmaceuticals (Lexington, KY).

### Cell Lines and Cell Culture

The human head and neck squamous cell carcinoma cell line, UMSCC10B was kindly provided by Dr. Thomas E. Carey (University of Michigan). Also used was the non-small cell lung cancer cell line A549, initiated by Giard et al [20]. These cell lines were cultured and maintained in DMEM supplemented with 10% fetal bovine serum and 1% penicillin streptomycin.

### Doxorubicin Treatment

A final doxorubicin concentration of 2.5 µg/ml was used in this study. Cells were incubated with doxorubicin for 1 hour at 37 C with shaking every 15–20 minutes. Then extrusion samples were placed in DMEM supplemented with 2% BSA for 3 hrs at 37degrees, again shaken every 15–20 minutes. No-extrusion samples were placed in Hanks buffer and placed on ice during duration of extrusion.

#### Flow Cytometry Analysis of Doxorubicin Efflux

Flow cytometric analysis of doxorubicin efflux was performed as in a previous study [21]. Cells were incubated with doxorubicin in Dulbecco's modified Eagle's medium containing 2% fetal calf serum (FCS). Afterwards, the cells were washed with Hanks' balanced salt solution and resuspended in doxorubicin-free culture medium. Cells were then allowed to extrude the drug for 2.5 hours at 37 C, except for no extrusion controls which were kept on ice, before analysis by flow cytometry. Dead cells were excluded by simultaneous staining with PI. Both Hoechst staining and doxorubicin efflux were analyzed using a fluorescence-activated cell sorter (FACSvantage SE, BD Biosciences, San Jose, CA) with CELLQuest (Largo, FL) software. The percentage difference in doxorubicin efflux was calcualted with the following formula: % change = [(CSC_no extrusion_– CSC_extrusion_) -(Control_no extrusion_– Control_extrusion_)]/(Control_no extrusion_– Control_extrusion_) * 100. The doxorubicin efflux is defined to be inversely related to the cellullar fluorescence measured by FACS.

### Western Blot Analysis

15 µg/mL or 30 µg/mL of CSC was added to cells 24 hours prior to lysing. Cells were lysed on ice for 10 minutes with buffer (0.1 M Tris, 2% SDS, 20% glycerin, and protease inhibitor tablets from Roche Diagnostics, Indianapolis, IN). Electrophoresis using 10% NuPage Bis-Tris gel separated proteins (20 ng per well), which were then transferred onto PVDF membrane. Membrane was blocked for one hour and incubated overnight in primary antibody diluted 1∶100 in blocking solution. Following addition of goat secondary antibody against mouse antibody (1∶1000 dilution in blocking solution), specific proteins were visualized using SuperSignal West Pico Luminol (Pierce, Rockford, IL). Band intensities were quantified by densitometry using the open-source software ImageJ (http://rsbweb.nih.govi/ij/) using a method outlined at http://lukemiller.org/index.php/2010/11/analyzing-gels-and-western-blots-with-image-j/.

### MTS Cell Viability Assay

MTS assay was performed using the CellTiter 96 AQueous non-radioactive cell proliferation assay (Promega, Madison, WI) following the manufacturer's instructions. Briefly, cells were trypsinized and plated in 100 µl of normal medium or CSC (15 µg/ml) medium in a 96-well format. After 24 hour incubation in 37C and 5% CO_2_, doxorubicin was added to the wells, followed by another 48 hours of incubation. Then, 20 µl of MTS reagent was added to each well followed by a 4 hour incubation period. A plate reader was then used to record the absorbance at 490 nm.

### Immunofluorescence

CSC was added to cells 24 prior to fixation. Cells were washed wish PBS and then fixed in 4% paraformaldehyde. 10% normal goat serum was used as the blocking agent. Primary antibody (diluted 1∶100) was then applied overnight in a humidified chamber. Secondary antibodies (diluted 1∶1000) Alexa Fluor 488 from Molecular Probes (Carlsbad, CA) or goat anti-mouse IgG from Chemicon International (Temecula, CA) were subsequently added to stain cells. Samples were washed successively with PBS, H_2_O, and ethanol. Stained cells were mounted and visualized using confocal microscopy.

### Flow Cytometry Analysis for Hoechst Efflux (Side Population Assay)

Cells were stained with 5 µg/mL of Hoechst 33342 (Sigma) in 4 mL of Dulbecco's modified Eagle's medium containing 2% bovine serum albumin (BSA) at 37°C for 90 min. After incubation, the cells were washed with Hanks' balanced salt solution and further incubated in 15 µg/ml or 30 µg/ml CSC for 24 hours. In addition, 2 µg/mL of propidium iodide (PI) was added for dead cell discrimination. Hoechst efflux was analyzed using a fluorescence-activated cell sorter (FACSvantage SE, BD Biosciences, San Jose, CA) with CELLQuest (Largo, FL) software. The area of the side population was determined using a separate sample (not shown) in which Hoecsht efflux in non CSC-treated cells was blocked by the drug verapamil. The region in which cells were lost upon treatment with verapamil was defined as the region that contained the putative side population, and was kept constant for all three samples (control, 15 ug/ml, 30 ug/ml).

## Results

### CSC treatment increases doxorubicin efflux via ABCG2

**Figure 1:**
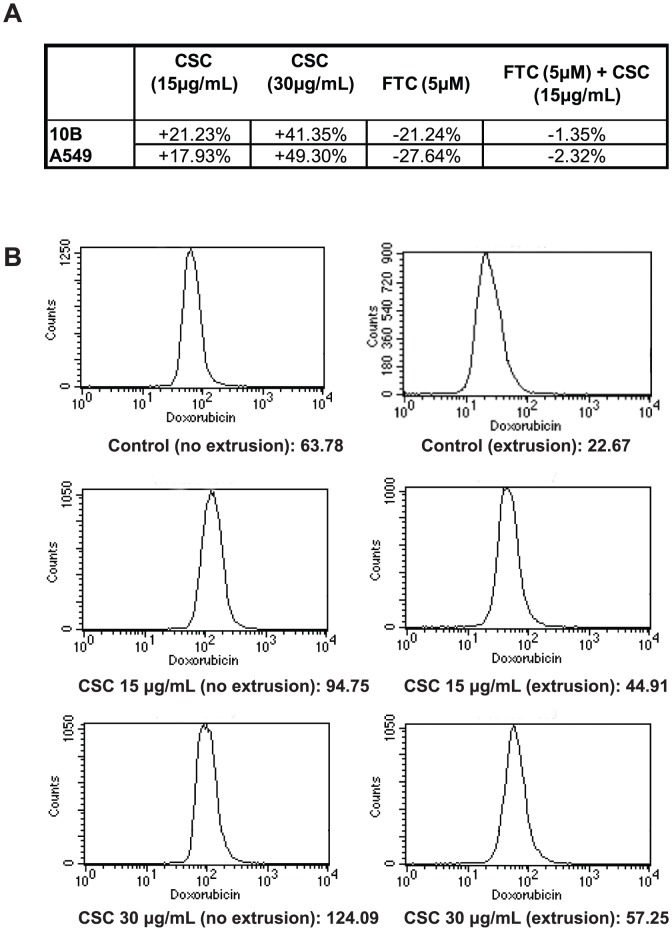
CSC treatment increases doxorubicin efflux in cell lines 10B and A549. (**A**) 15 and 30 µg/mL cigarette smoke condensate (CSC) increases doxorubicin efflux in both cell lines 10B and A549 in a dose-dependent pattern. The specific ABCG2 inhibitor fumitremorgin C (FTC) is able to reverse the CSC-mediated increase in doxorubicin efflux. Percentage change was derived from flow cytometric analysis of median intracellular doxorubicin levels and calculated using the formula described in the methods section. (**B**) CSC increases doxorubicin efflux dose-dependently. Relative efflux gauged by comparing the differences in the median doxorubicin fluorescence channels of extrusion and no extrusion samples. Numerical label reflect the median channel of doxorubicin fluorescence as measured in one sample of flow cytometric analysis of intracellular doxorubicin levels in 10B. Although absolute fluorescence levels in the treated samples are higher due to fluorescence of CSC, the decrease from no extrusion to extrusion is greater for these samples compared to control.

To determine whether CSC treatment could regulate drug transporter activity, we first measured the changes in doxorubicin extrusion observed upon addition of CSC. The doses of CSC (15 ug/ml, 30 ug/ml) were chosen based on doses used in previous studies [22], [23], [24]. Intracellular doxorubicin levels were determined by using flow cytometry to measure the drug's fluorescence in individual cells. An optimal extrusion time frame (2.5 hours) was selected to balance the effects of cytotoxicity against detectable changes in doxorubicin efflux. In order to account for background fluorescence caused by CSC, differences in median fluorescence between an extrusion sample (kept at 37C) and no extrusion sample (kept on ice) were compared rather than absolute fluorescence levels. Results indicated that CSC increased doxorubicin efflux in a dose-dependent fashion (Figure 1a,b). A specific inhibitor for ABCG2 transport activity, Fumitremorgin C (FTC), was introduced to cells to be examined by the doxorubicin efflux assay. Addition of FTC reversed the effect of CSC, suggesting that ABCG2 is the transporter responsible for CSC-induced doxorubicin efflux. The data presented in this figure are representative of at least two independent trials.

### CSC induces membrane localization and expression of ABCG2

**Figure 2:**
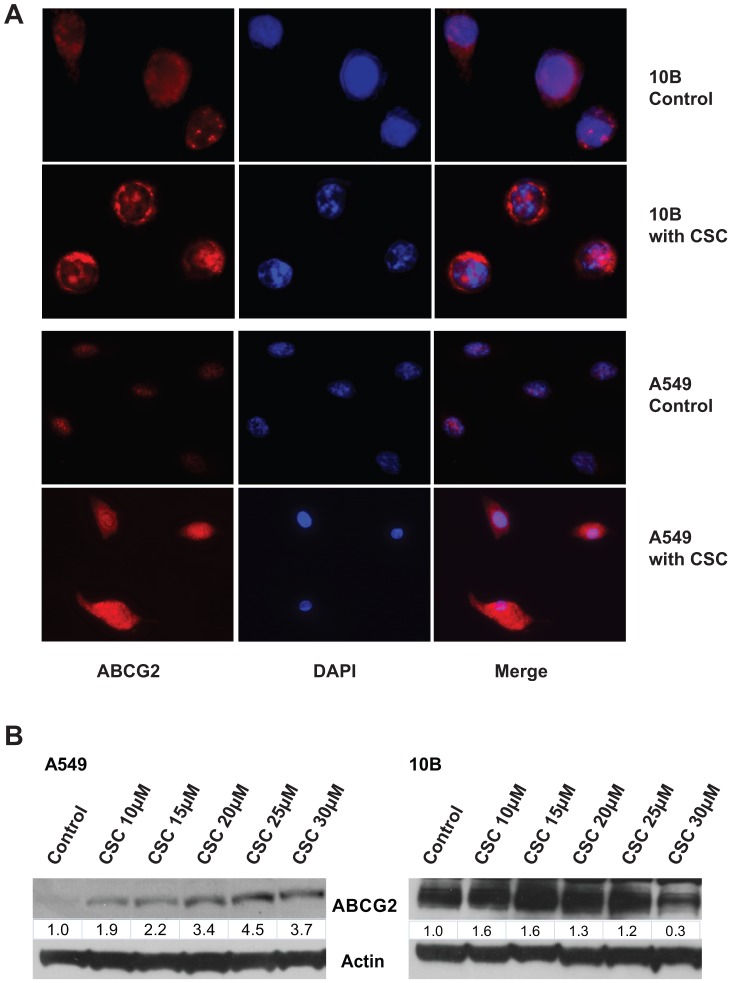
CSC treatment induces the expression and membrane localization of ABCG2. (**A**) Immunofluorescence microscopy identifies the cellular localization of ABCG2 with and without CSC. Results indicate that CSC exposure induces translocation of ABCG2 to the plasma membrane in 10B and A549. (**B**) Western blot analysis shows dose dependent increase in ABCG2 expression in response to CSC treatment.

**Figure 3:**
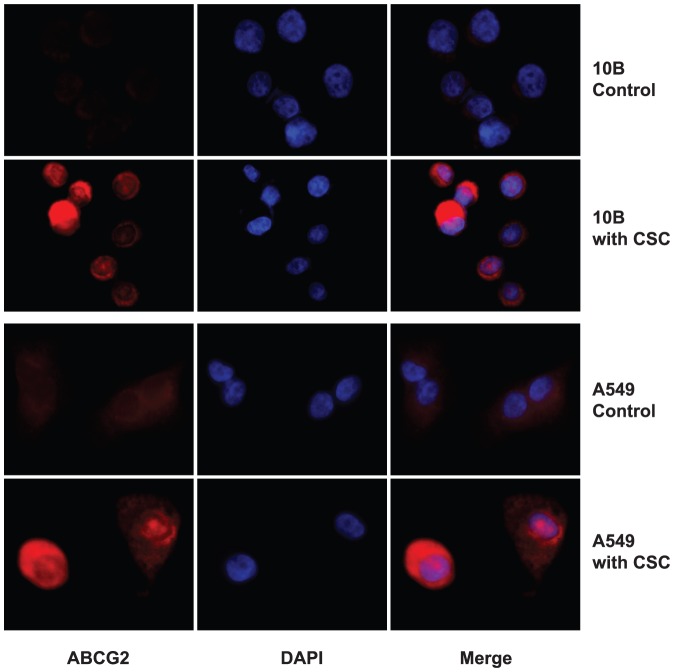
CSC treatment intensifies ABCG2 staining in non-permeabilized cells. Immunofluorescence was repeated on non-permeabilized cells to demonstrate the increase in membrane concentrations of ABCG2. Results indicate weak staining of ABCG2 on the surface of non-treated cells, while much stronger staining was observed on the surface of CSC treated cells.

Only ABCG2 localized in the plasma membrane are able to efflux doxorubicin. To verify that CSC promotes drug resistance by regulating ABCG2 activity, an immunofluorescence experiment was performed to determine the effect of CSC on ABCG2 localization. Photographs comparing control cells to CSC-treated cells showed that CSC increased the plasma membrane concentrations of ABCG2 (Figure 2a). Interestingly, cytoplasmic protein levels of ABCG2 were not visibly lower following translocation, suggesting that CSC perhaps also upregulates total levels of ABCG2. Indeed, western blotting of whole-cell lysate revealed that CSC also increased the total expression of ABCG2 in a dose-dependent manner (Figure 2b). ABCG2 expression was lower in the highest dose, most likely as a result of CSC cytotoxicity. Immunofluorescence assays were also repeated with non-permeabilized cells in order to further visualize the translocation of ABCG2, since only proteins on the cell surface will be stained in this case. Consistent with our hypothesis, ABCG2 staining was barely visible in non-permeabilized control cells, but was clearly present on the surface of CSC-treated cells (Figure 3). Photographs shown are representative of at least two independent trials.

### CSC-induced doxorubicin efflux and ABCG2 translocation are mediated by PI3K/Akt signaling

**Figure 4:**
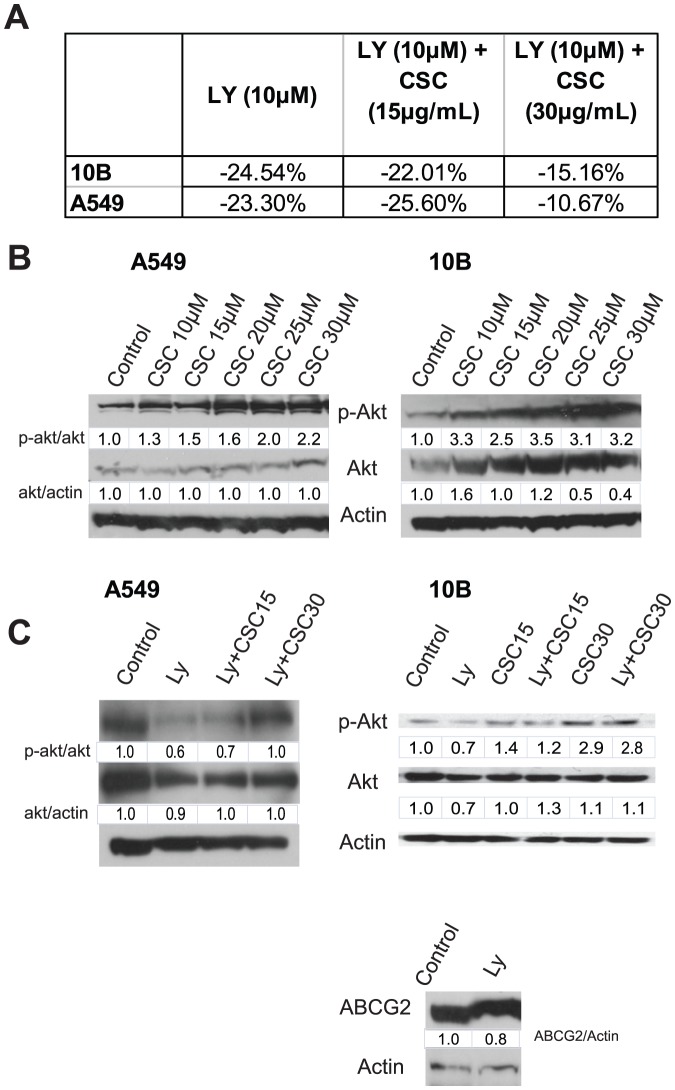
Inhibition of PI3K reduces CSC-mediated doxorubicin efflux. (**A**) PI3K inhibitor LY294002 (LY) decreases doxorubicin efflux in cell lines 10B and A549. Values are derived from flow cytometric analysis of intraceulluar doxorubicin levels, and are computed relative to non-LY treated controls. (**B**) Western blot analysis showing the change in levels of phospho-Akt and Akt with increasing dosage of CSC. (**C**) Western blot analysis showing the reversal of Akt activation by treatment with LY, and the expression of ABCG2 in the presence of LY.

We have previously shown that Akt regulates the function of ABCG2 in HSNCC [25]. We therefore hypothesized that CSC was promoting doxorubicin efflux through the activation of Akt. To test this hypothesis, we re-analyzed the rate of doxorubicin efflux in the presence of the PI3K inhibitor LY294002. The addition of LY reversed the CSC-induced increase in doxorubicin efflux (Figure 4a). Western blot analysis revealed that CSC increased the levels of phospho-Akt dose-dependently (Figure 4b). As expected, addition of the PI3K inhibitor LY294002 blocked CSC-induced activation of Akt (Figure 4c). Interestingly, treatment with LY alone did not significantly decrease the levels of ABCG2 relative to control (Figure 4c). Together, these results show that PI3K/Akt is one of the key mechanisms involved in regulation of ABCG2 function, but may not be critically involved in modulating ABCG2 expression.

### CSC treatment increases mitoxantrone efflux

**Figure 5:**
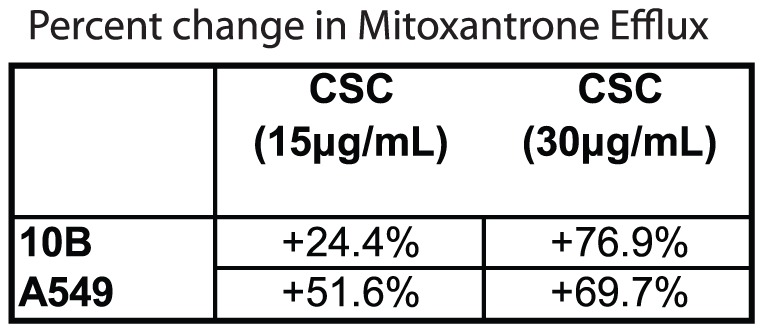
CSC treatment increases rate of mitoxantrone efflux. Flow cytometric analysis of intracellular mitoxantrone levels revealed significantly higher rates of extrusion in CSC-treated cells. Values shown are the percentage differences compared to non-treated samples.

If CSC-mediated doxorubicin resistance definitively involved ABCG2, then CSC should also be able to protect against other ABCG2 substrates. Thus, we tested whether CSC could also enhance the cellular efflux of mitoxantrone, a substrate of ABCG2 [26]. Consistent with our proposed mechanism, CSC significantly increased the cellular efflux of mitoxantrone, confirming the involvement of ABCG2 (Figure 5).

### CSC treatment promotes cell viability during doxorubicin exposure

**Figure 6:**
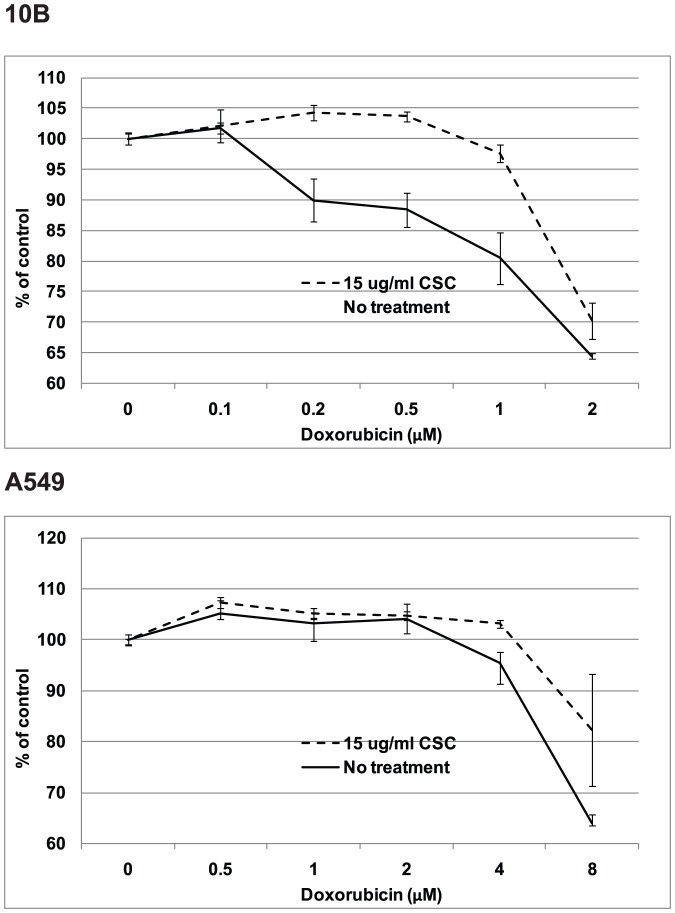
CSC treated cells show enhanced viability during doxorubicin exposure. MTS cell viability assays testing the viability of control vs. CSC-treated 10B and A549 cells in the presence of doxorubicin. Absorbance (Y-axis) values were normalized by dividing over the absorbance of the 0 µM doxorubicin samples. Results indicate that CSC has a protective effect against doxorubicin-induced cell death. Error bars represent SEM.

Although CSC clearly induces greater drug efflux, we asked whether this translated to a detectable change in drug resistance. An MTS assay, which measures the proportion of viable cells, was performed to compare the survival curves between control cells and CSC-treated cells in the presence of increasing doxorubicin concentration (Figure 6). In order to account for discrepancies in cell proliferation, all absorbances values in a sample were divided by the absorbance of their respective 0 µM doxorubicin controls. Results showed that a significantly higher proportion of CSC-treated cells remained viable compared to non-treated cells, suggesting that CSC ultimately protects cells from doxorubicin-induced death. Figure 6 shows representative experiments performed in triplicates.

### CSC treatment increases the side population

**Figure 7:**
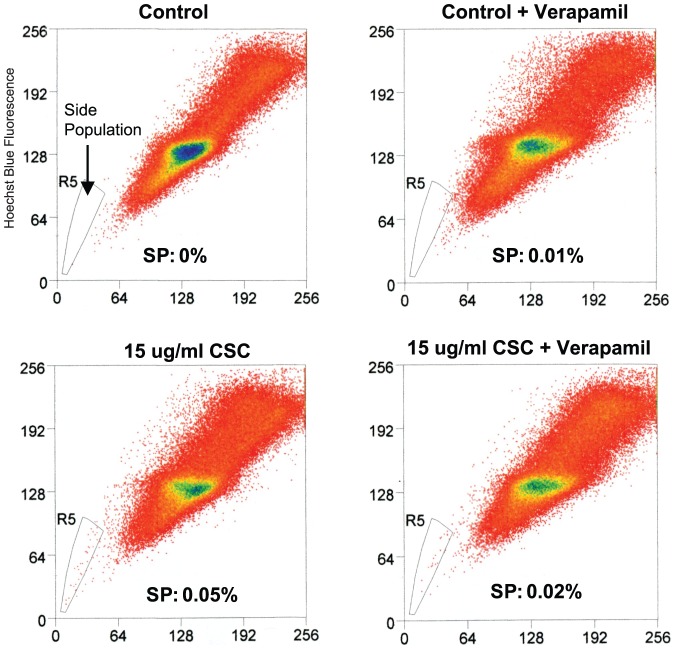
CSC treatment increases the side population in 10B cells. FACS-based Hoechst extrusion assay shows the size of side population (SP) in 10B cells incubated with two doses of CSC. The size of the SP increases with increasing dosage of CSC. Cells were incubated in 5 ug/ml of Hoechst for 90 min and then in DMEM w/CSC or normal DMEM for 24 hours.

Side population (SP) cells are defined as cells that show higher efflux of Hoechst 33342 dye, a specific substrate for ABCG2, relative to the main population. We tested whether CSC could increase the size of the SP in 10B cells. Indeed, a Hoechst-extrusion assay revealed that the size of the SP was greater in CSC-treated cells compared to non-treated cells (Figure 7). In the presence of verapamil, the increase in SP was partially abrogated, suggesting that ABCG2 is at least partially responsible for the increase in side population, although it remains possible that other drug pumps could play a role in CSC-induced side population. These results confirms the involvement of ABCG2 in CSC-induced drug resistance and suggest the possibility that CSC could regulate cancer stem cell properties, since SP cells have been shown to be much more chemoresistant and tumorigenic than non-SP cells [15], [19], [27].

### Nicotine treatment promotes cell viability during doxorubicin exposure

**Figure 8:**
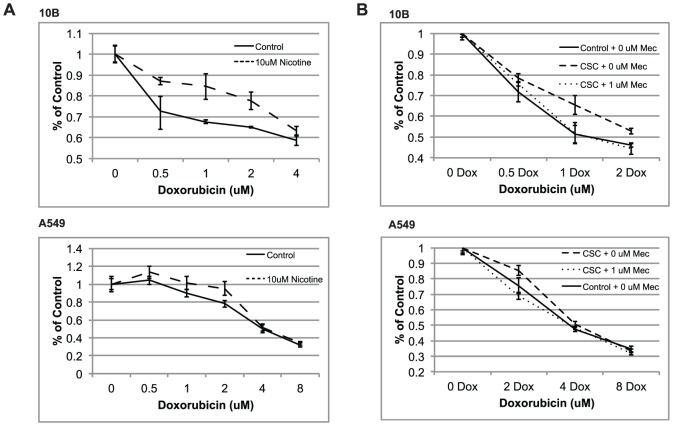
Nicotine-treated cells show enhanced viability during doxorubicin exposure. (**A**) MTS proliferation assay testing the viability of control vs. nicotine-treated cells in the presence of doxorubicin. Absorbance (Y-axis) values were normalized by dividing over the absorbance of the 0 µM doxorubicin samples. (B) MTS viability assays demonstrating the reversal of CSC-induced doxorubicin resistance by the nAChR inhibitor mecamylamine. Taken together, these results indicate that nicotine is at least partially responsible for the protective effect of CSC against doxorubicin-induced cell death. Error bars represent SEM.

Because cigarette smoke condensate is a mixture of a myriad of compounds, it would be useful to determine the specific compound within CSC that could be responsible for promoting drug resistance. We therefore aimed to determine whether nicotine, a specific component of CSC, could be responsible for CSC-induced drug resistance. An MTS assay was performed to compare survival curves between control cells and 10 µM nicotine-treated cells in the presence of increasing doxorubicin concentration (Figure 8a). The results of this experiment showed that nicotine provided a significant advantage in cell viability during exposure to doxorubicin. To confirm that nicotine is important in CSC-induced drug resistance, an MTS assay was performed to determine whether mecamylamine, a non-selective nicotinic acetylcholine receptor inhibitor, could reverse the drug-protective effect of CSC. Results demonstrated a survival advantage in CSC-treated cells that was mitigated upon treatment with 1 µM mecamylamine (Figure 8b).

## Discussion

Although previous studies have shown that CSC has the potential to promote chemoresistance [18], [28], [29], identifying its mechanism of action remains an important finding. Our data supports a model wherein CSC activates Akt to induce the translocation of ABCG2 to the plasma membrane, thereby enhancing drug extrusion and chemoresistance. We also observed that CSC is able to induce the expression of ABCG2 in addition to regulating translocation. Interestingly, at the highest dose of CSC (30 µM), ABCG2 expression was lowered perhaps due to cytotoxicity, although doxorubicin efflux remained high. This suggests that ABCG2 translocation alone is more than sufficient to account for CSC-induced doxorubicin efflux.

While the involvement of Akt in ABCG2 translocation is well-supported by our data and by the work of others [30], [31], it is unclear whether CSC-induced expression of ABCG2 is also mediated by Akt. Our western blot data (Figure 4C) seems to suggest that CSC may regulate the expression of ABCG2 independently of PI3K/Akt signaling. This is consistent with the observations of Bleau et al, who found that Akt regulates the function (i.e. translocation) of ABCG2, but not its expression [32]. A recent study showed that aryl hydrocarbon receptor (Ahr) is a direct transcriptional activator of ABCG2 in breast cancer [33]. Benzo[α]pyrene (B[a]P), one of the key carcinogens in cigarette smoke, is a potent activator of Ahr [34]. Thus, it is plausible that B[a]P binds to Ahr, which then activates the transcription of ABCG2 and accounts for the observed increase in ABCG2 protein. Another possibility is that CSC acts through microRNA regulation to increase ABCG2 protein levels. CSC has been shown to modulate the expression of a variety of microRNAs [35], [36]. It is possible that miR-519c, which has been shown to repress the translation of ABCG2 [37], is included among the microRNAs that are downregulated by exposure to CSC. Although these theories have yet to be confirmed by future work, what is clear is that ABCG2 is a key component of CSC-induced chemoresistance, making it a potential therapeutic target for smoking-induced cancers. Treatments combining the administration of ABCG2 inhibitors and conventional chemotherapy may prove effective in eradicating both the bulk and chemoresistant populations of a tumor, thereby reducing the likelihood of recurrence.

We observed that CSC has the ability to expand the side population. By definition, the cells within a population that display higher efflux of DNA-binding dye Hoechst 33342 via ABCG2 constitute the SP. In multiple cancers these cells have been observed to be more cancer stem cell-like in nature, i.e. they are more tumorigenic than non-SP cells. In theory, cancer stem cells are responsible for disease recurrence because of their ability to survive chemotherapy and thereafter recapitulate the original tumor through self-renewal and differentiation. The observation that SP cells show higher efflux of DNA-binding dye and exhibit stem cell-like behavior is consistent with the idea that cancer stem cells should constitute the chemoresistant population within a tumor. There has also been evidence to suggest that cancer stem cells are responsible for invasion and metastasis. Thus, CSC-induced expansion of the SP could serve as evidence of a potentially novel paradigm in which cigarette smoke induces cancer by promoting a cancer stem cell phenotype within existing tumor cells. Initially, these cells are benign and highly differentiated, but obtain a malignant, undifferentiated “cancer stem cell” phenotype upon sustained exposure to cigarette smoke. Further investigation into this model would provide critical understanding of how smoking initiates cancer.

Although some effects of CSC warrant further investigation, the implications of this study remain clear: cancer patients who continue to smoke cigarettes during chemotherapy may stand to bear a much poorer prognosis. Therefore, smoking cessation during treatment may play a major role in maximizing the likelihood of success. Furthermore, we have identified nicotine as a mediator of the drug resistance-promoting effect of CSC. This finding suggests that nicotine, a compound that has been implicated in oncogenesis may also have a role in drug resistance, possibly through its regulation of ABCG2. It is important to further investigate the detailed mechanisms which govern CSC-mediated ABCG2 expression, and to assess the effectiveness of ABCG2 as a potential drug target. It would also be worthwhile to determine other effects of CSC that may aid cancer progression, especially those which target cancer stem cell properties.
